# Obesity and Heart Failure: Introducing the Theme

**DOI:** 10.3390/jcdd13040153

**Published:** 2026-03-30

**Authors:** Francesco Monitillo, Paolo Basile, Giuseppe Lisco

**Affiliations:** 1 Cardiology University Unit, University of Bari Aldo Moro, Piazza Giulio Cesare 11, 70124 Bari, Italy; dr.francescomonitillo@gmail.com; 2 Unit of Cardiology, San Pio Hospital, Via del Mercato, 74011 Castellaneta, Italy; paolo.basile@uniba.it; 3 Interdisciplinary Department of Medicine, School of Medicine, University of Bari “Aldo Moro”, Piazza Giulio Cesare 11, 70124 Bari, Italy

**Keywords:** obesity, excess adiposity, heart failure, preserved ejection fraction, GLP-1RA, SGLT2i

## Abstract

Obesity is a chronic, highly prevalent disease affecting nearly one-third of the global population and represents a major independent risk factor for heart failure (HF), particularly heart failure with preserved ejection fraction (HFpEF). Excess adiposity—especially visceral and epicardial adipose tissue (EAT)—acts as an active endocrine and immune organ, promoting chronic low-grade inflammation, oxidative stress, endothelial dysfunction, and adverse myocardial remodeling. Expanded EAT exerts both paracrine inflammatory effects and mechanical constraint on the myocardium, contributing to diastolic dysfunction, microvascular impairment, atrial arrhythmogenesis, and elevated filling pressures despite preserved systolic function. Evidence demonstrates a dose–response relationship between increasing body mass index and incident HF. Clinically, obesity-related HFpEF is characterized by concentric left ventricular hypertrophy, impaired relaxation, increased plasma volume, reduced exercise tolerance, and relatively low natriuretic peptide levels, complicating diagnosis. HF management includes traditional treatment with diuretics, renin-angiotensin system inhibitors, β-blockers, mineralocorticoid receptor antagonists, and angiotensin receptor-neprilysin inhibitors. These agents widely remain foundational as they primarily target hemodynamic and neurohormonal pathways in HF. In contrast, sodium–glucose cotransporter 2 inhibitors consistently reduce HF hospitalizations across the ejection fraction spectrum, while glucagon-like peptide-1 receptor agonists and dual incretin therapies (e.g., tirzepatide) promote substantial weight loss, improve symptoms, and demonstrate promising anti-remodeling effects in obesity-related HFpEF. Recognizing obesity-driven HF as a distinct cardiometabolic entity supports an integrated therapeutic strategy combining structured weight reduction with guideline-directed HF polypharmacotherapy to address both hemodynamic burden and upstream adiposity-related mechanisms.

## 1. Introduction

Obesity is a chronic widespread condition characterized by excess adiposity with or without abnormal distribution or function of the adipose tissue associated or not to obesity-related comorbidities [1]. Estimates indicate a worldwide obesity prevalence of around 30% [2].

Obesity markedly increases the risk of cardiovascular diseases (CVD) as the consequence of intricate pathophysiological interconnections between adipose tissue dysfunction, obesity-related comorbidities and complications (Table 1) [3].

Heart failure (HF) is tightly related to obesity and excess adiposity burdens approximately 20% of patients with established diagnosis of HF, surging to over 80% in HF with preserved ejection fraction (HFpEF) subset [4].

## 2. Pathogenetic Role of Excess Adiposity, Visceral Depots, and Epicardial Fat in Obesity-Driven Myocardial Injury

Adipose tissue is recognized as an active endocrine and immune organ. Expanded adiposity drives chronic low-grade inflammation and dysregulated adipokine profiles. Oxidative stress-generated by inflamed visceral adipose depots directly disrupts vascular homeostasis due to several events, such as production of reactive oxygen species, nitric oxide bioavailability, dysfunctional perivascular adipose tissue dysregulation, and systemic inflammation further injures the endothelium [5].

The metabolic milieu of obesity—dyslipidemia with small dense LDL, hypertriglyceridemia, and insulin resistance—accelerates all phases of atherosclerosis, from intimal lipid accumulation to plaque progression and instability [6].

Excess weight also increases blood volume and cardiac workload, activates neurohormonal systems, increases arterial pressure levels, and promotes left ventricular hypertrophy and remodeling [7]. 

Epicardial adipose tissue (EAT) can exert paracrine effects that worsen diastolic function [8]. Epicardial adipocytes and infiltrating immune cells—activated macrophages (M1 phenotype) and pro-inflammatory T-lymphocytes—produce tumor necrosis factor-α (TNF-α), interleukin-6 (IL-6), interleukin-1β (IL-1β), and monocyte chemoattractant protein-1. At the same time, local expression of adiponectin is significantly reduced with negative effects on inflammation, myocardial fibrosis and vasoconstriction [9]. This imbalance promotes established low-grade inflammation in the adjacent myocardium, facilitating extracellular matrix deposition and interstitial fibrosis. The activation of inflammatory signaling pathways, including nuclear factor kappa B and the inflammasome, further amplifies local oxidative stress and cellular dysfunction. Local release of inflammatory mediators and reactive oxygen species also impairs endothelial nitric oxide bioavailability, leading to coronary endothelial dysfunction and microvascular rarefaction. All these alterations contribute to myocardial ischemia in the absence of obstructive coronary artery disease, a feature frequently observed in obesity-related heart disease and HFpEF [10]. EAT expansion also imposes mechanical constraints on the myocardium. As epicardial fat volume increases, the tissue exerts external compressive forces on the myocardium, effectively reducing ventricular compliance. This mechanical restraint interferes with normal diastolic relaxation and filling, resulting in elevated intracardiac filling pressures even when ventricular volumes are preserved. Such effects are particularly pronounced at the left ventricular level and are considered a key contributor to the diastolic dysfunction typically characterizing HFpEF [11]. The mechanical and inflammatory actions of EAT are relevant in the atrial myocardium, as epicardial fat infiltration and fibrotic remodeling create structural and electrical heterogeneity disrupting normal conduction pathways. This process provides a favorable substrate for atrial arrhythmias—such as atrial fibrillation—which is highly prevalent in individuals with overweight and obesity [12].

Excess adiposity also triggers hyperdynamic circulation and stroke work demands, thus forcing chronic preload-to-afterload mismatch, elevated filling pressures, eccentric ventricular hypertrophy, lastly culminating in diastolic failure. Ectopic fat deposition (visceral, hepatic, and myocardial) induces lipotoxicity through ceramide and sphingolipid overload that impairs mitochondrial β-oxidation and myocyte injury. Obesity also disrupts the cardiac stem progenitor cell (CPC) niche and inhibits CPC differentiation into cardiomyocytes, ultimately depleting regenerative reserves and perpetuating maladaptive remodeling [13] (Table 2).

## 3. Clinical Evidence of Obesity Impact on HF and Related Prognosis

Obesity has emerged as a relevant and independent risk factor for the development of HF. Longitudinal analyses from a general population cohort reveal a positive association between incremental increases in body mass index (BMI) and the risk of new-onset HF. Specifically, each 1 kg/m^2^ increment in BMI confers a 6% increase in HF incidence independent of canonical cardiovascular (CV) risk factors, confirming an exposure–response relationship in observational settings. Moreover, higher BMI is also associated with increased HF-specific (4%) and all-cause mortality (1%) in these cohorts [14]. A meta-analysis of 25 cohort studies found a summary HF relative risk of 42% for each 5 kg/m^2^ increment in BMI, 28% for 10 cm higher waist circumference, and 33% per 0.1-unit higher waist–hip ratio. Pooled estimates reported significant positive association between HF risk and both visceral and pericardial fat (8%). These findings are even stronger in HFpEF than HF with reduced ejection fraction (HFrEF) [15]. Data collected from a National U.S.A. database, including more than 1 million patients, indicates obesity (odds ratio 1.84; 95% CI: 1.22–2.76) and morbid obesity (odds ratio 1.81; 95% CI: 1.22–2.70) increased the risk of in-hospital mortality compared with normal weight, and was also associated with higher odds of in-hospital mortality in both HFrEF and HFpEF [16].

Additional anthropometric parameters, such as waist circumference and waist-to-hip ratio (WHR), reflect cardiometabolic risk independently of BMI as expression of abdominal fat distribution and visceral adiposity strongly. WHR has been shown to outperform or complement BMI in predicting metabolic and cardiovascular outcomes, supporting its use as a practical clinical biomarker of metabolically hazardous obesity [17,18].

Sarcopenic obesity, as defined by EASO and ESPEN, combines excess adiposity with reduced muscle mass and/or function, assessed by integrating BMI or waist circumference, functional measures, and body composition [19]. Compared with obesity alone, sarcopenic obesity induces a synergistic worsening of insulin resistance, atherogenic dyslipidemia, and pro-inflammatory status, driven by both visceral fat excess and lower metabolically active muscle mass [20]. Consequently, sarcopenic obesity conveys a substantially higher cardiovascular risk, with increased atherosclerosis, arterial stiffness, HF, and cardiovascular mortality [21]. Nowadays, estimations suggest that up to 40% of patients with excess weight may have sarcopenic obesity, an epidemiological proportion that assumes considerable relevance given the amplified cardiometabolic risk associated with this phenotype.

Among patients with established HF, overweight or obese individuals often exhibit lower all-cause and CV mortality and, in some cohorts, reduced rates of HF-related hospitalization, compared with normal-weight or underweight patients [22]. This phenomenon is also known as the so-called obesity paradox [23]. From an observational standpoint, evidence demonstrates a U- or J-shaped relationship between BMI and mortality in HF populations; so, while underweight, low-normal BMI categories, and class III obesity are associated with the highest mortality risk, overweight and class I–II obesity are associated with the lowest hazard ratios for death after multivariable adjustments [24]. Several large registries report that overweight and moderately obese HF patients experience lower rates of rehospitalization and shorter lengths of hospital stay when admitted for acutely decompensated HF, potentially reflecting greater metabolic reserve and tolerance to acute catabolic stress. However, data are heterogeneous highlighting the context-dependent nature of the association. Thus, while mortality outcomes often favor higher BMI categories, hospitalization risk does not consistently follow the same pattern [25].

Mechanistically, the obesity paradox is unlikely to reflect protective effects of excess adiposity but, instead, it is best explained by a combination of methodological biases and biological confounders. First, BMI alone fails in distinguishing between fat mass and fat-free mass and does not provide information about overall body composition and visceral adiposity. Patients with high BMI may have greater skeletal muscle mass, which is independently associated with improved HF prognosis [26] or fluid retention [27]. Conversely, lower BMI in HF often reflects cardiac cachexia, sarcopenia, or advanced disease severity, all strong predictors of mortality especially in patients with HFpEF [28]. Confounder factors and markers of disease severity also reflect HF progression better than BMI. Advanced HF patients frequently experience unintentional weight loss and sarcopenia, which are independent markers of disease severity and independently predict poor outcomes. So, overweight or mildly obese HF patients may simply represent a group with less severe metabolic and catabolic stress, biasing survival comparisons. Additional contributors include lead time and selection biases, mostly driven by the fact that patients with excess weight frequently develop HF at a younger age and are consequently prone to be treated prior and more intensively compared to normal- or underweight individuals [29].

## 4. Characterization of Clinical, Laboratory, and Echocardiographic Hallmarks of Obesity-Associated Heart Failure

Obesity-driven HF represents a distinct clinical phenotype characterized by well-defined structural, functional, and metabolic abnormalities. The main phenotype is severe or long-standing obesity (BMI ≥ 35 kg/m^2^), with a higher prevalence among women than men, and frequent coexisting comorbidities such as arterial hypertension, type 2 diabetes (T2D), and obstructive sleep apnea. This combination generates a high cardiometabolic burden, and early recognition of HF is often hampered by symptom overlapping with obesity (e.g., fatigue and dyspnea) [30].

Hemodynamically, patients exhibit expanded plasma volume and elevated cardiac output, especially in early phases of HF, along with increased cardiac preload and afterload, and arterial stiffness. These changes contribute to progressive left ventricular remodeling, characterized by concentric hypertrophy, increased left ventricle mass index, and relative wall thickening, often with preserved or mildly reduced cavity size. Diastolic dysfunction is a hallmark, with impaired relaxation, elevated E/e′ ratio, and reduced diastolic reserve, resulting in elevated filling pressures that become particularly apparent during physical efforts [31]. Left atrial enlargement and impaired reservoir function reflect persisting elevated pressures, while right ventricular involvement and elevated pulmonary artery pressures contribute to exercise intolerance and congestion in more advanced stages. EAT is increased in thickness and exerts both mechanical constraints and paracrine inflammatory effects, which may further impair ventricular compliance, promote cardiac remodeling, and significantly impair long-term outcomes [32].

Functionally, these patients often display reduced cardiometabolic fitness—expressed as VO_2_ max—chronotropic impairment, and abnormal ventricular–vascular coupling, which together explain disproportionate exertional dyspnea relative to resting findings. Biomarkers may be misleading: natriuretic peptide levels are frequently lower than expected for the degree of congestion (renal hyperfiltration), while inflammatory markers such as CRP are elevated, posing diagnostic challenges [33] (Table 3).

**Figure 1:**
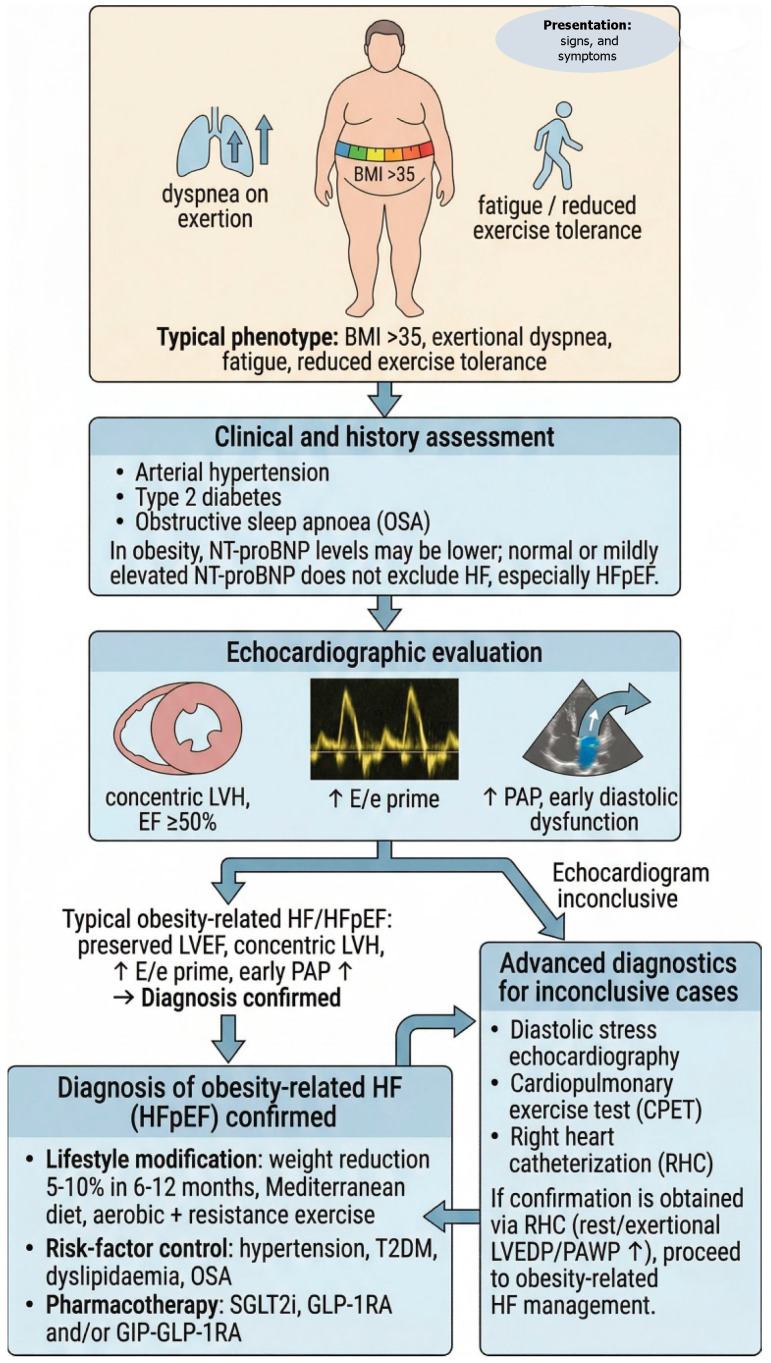
This presents a stepwise diagnostic and management algorithm for obesity-associated heart failure, a phenotype predominantly characterized by HFpEF. It emphasizes careful clinical assessment, cautious interpretation of natriuretic peptides, comprehensive echocardiographic evaluation, and functional testing when needed to confirm elevated filling pressures. Management integrates guideline-directed heart failure therapy with aggressive comorbidity optimization and structured weight reduction, recognizing obesity as a central driver of the syndrome [30,31,32,33,34]. List of Abbreviations: BMI, Body Mass Index; HF, Heart Failure; HFpEF, Heart Failure with Preserved Ejection Fraction; LVEF, Left Ventricular Ejection Fraction; LVH, Left Ventricle/Left Ventricular Hypertropia; E/e, ratio of Early mitral inflow velocity to early diastolic mitral annular velocity; PAP, Pulmonary Artery Pressure; NT-proBNP, N-Terminal pro-B-type Natriuretic Peptide; T2DM, Type 2 Diabetes Mellitus; OSA, Obstructive Sleep Apnea; SGLT2i, Sodium–Glucose Cotransporter type 2 inhibitors; GLP-1RA, glucagon-like peptide-1 receptor agonists; GIP, glucose-dependent insulinotropic polypeptide; CPET, CardioPulmonary Exercise Test; RHC, Right Heart Catheterization; LVEDP, Left Ventricular End-Diastolic Pressure; PAWP, Pulmonary Artery Wedge Pressure. ↑, increase.

Collectively, this phenotype manifests as obesity-driven HFpEF, marked by high symptom burden, exercise intolerance, and variable edema, emphasizing the need for careful cardiometabolic and hemodynamic evaluation (Figure 1).

HFpEF diagnosis requires a structured and multiparametric approach integrating clinical evaluation, laboratory testing, and cardiac imaging [34]. To standardize and facilitate this complex diagnostic process, several scoring systems have been developed in recent years. Among the most widely adopted are the Heart Failure Association Pretest Probability of Heart Failure with Preserved Ejection Fraction (HFA-PEFF) [35] and the heavy, hypertension, atrial fibrillation, pulmonary hypertension, elder, and filling pressures (H2FPEF) [36]. These algorithms incorporate a combination of clinical variables, laboratory data—including natriuretic peptide concentrations—and echocardiographic parameters to improve diagnostic discrimination between HFpEF and non-cardiac causes of dyspnea. In addition to supporting clinical decision-making, these tools help guide further diagnostic testing, such as diastolic stress echocardiography or invasive hemodynamic assessment, in cases where diagnostic uncertainty persists [37]. Importantly, beyond their diagnostic utility, these scoring systems also provide incremental prognostic information. They are capable of identifying individuals without a formal diagnosis of HFpEF who are nonetheless at increased risk for future HF-related events, thereby highlighting a subgroup that could benefit from closer surveillance and early interventions [38]. Although methodological differences exist between HFA-PEFF and H2FPEF scores—reflecting distinct weighting of clinical versus imaging parameters—their overall diagnostic performance in unselected patient populations has been shown to be satisfactory (Table 4) [39]. However, data regarding their performance in specific phenotypic subgroups, particularly in patients with obesity, remain limited. This gap is clinically relevant, given the strong epidemiological and pathophysiological link between obesity and HFpEF. In individuals with obesity, several factors complicate the diagnostic workflow. First, typical symptoms—such as exertional dyspnea and reduced exercise tolerance—may overlap with or be attributed to obesity itself, increasing the risk of both over- and under-diagnosis. Second, circulating natriuretic peptide levels are consistently lower in individuals with excess weight compared with normal-weighted counterparts, even in the presence of elevated filling pressures, thereby potentially reducing the sensitivity of biomarker-based diagnostic criteria. Third, excess adipose tissue may impair echocardiographic image quality by attenuating ultrasound transmission, limiting accurate assessment of diastolic parameters and structural remodeling [40,41,42].

## 5. Pharmacological Modulation of Volume Overload and Cardiac Remodeling: Mechanisms and Evidence of Conventional Treatments

Obesity is characterized by a wide spectrum of pathophysiological processes profoundly impacting on cardiac pump and prompting HF. These alterations include sodium-water retention, neurohormonal activation, myocardial hypertrophy, interstitial fibrosis, and dysfunctional left ventricular remodeling. Therapeutic strategies targeting these mechanisms remain foundational in the management of obesity-related HFpEF (Table 5).

Diuretics—including loop and thiazide/thiazide-like agents—are the foremost pharmacological intervention to relieve congestion via natriuresis and diuresis, reducing intravascular volume and venous congestion [43]. Loop diuretics, such as furosemide and torasemide, act on the Na^+^-K^+^-2Cl^−^ cotransporter in the thick ascending limb of the Henle’s loop, resulting in an imbalanced countercurrent gradient formation that impairs renal concentration capability in response to antidiuretic hormones and produces rapid and potent diuresis [44]. While, in acutely decompensated HF, loop diuretics are most effective in symptom relief and hemodynamics improvement [45], their long-term effects on hemodynamic and hard endpoints remain neutral or controversial when compared to guideline-directed neurohormonal therapies. In fact, chronic administration of loop diuretics may reduce intrarenal plasma flow, consequently impairing glomerular filtration rate [46]. Also, loop diuretics activates the renin–angiotensin–aldosterone system that, in turn, activates compensative mechanisms of sodium and water retention and potentially counteract favorable remodeling effects on cardiomyocytes [47]. Although direct clinical trials detailing obesity-related HFpEF management are lacking, evidence seems to indicate that chronic exposure to loop diuretics—especially with high doses—is associated with worse clinical outcomes in HFpEF, including excess of CV mortality [48]. Patients with obesity are particularly responsive to intravenous loop diuretic treatment although being predisposed to a higher risk of worsening renal function following natriuresis [49]. Nevertheless, loop diuretics are the first therapeutic option—with almost comparable efficacy between furosemide and torsemide—in HFpEF hospital management [50] and acetazolamide—as add-on to background treatment—usually potentiate the decongestive effects of loop diuretics in acute decompensated HF [51]. Thiazide and thiazide-like diuretics act at the distal convoluted tubule inhibiting the sodium-chloride cotransporter, accentuating residual sodium excretion also impairing aldosterone-mediated sodium resorption efficiency [52]. These agents do not influence cardiac structural remodeling and have mild or long-term effects on HFpEF and overall CV outcomes [53]. Sequential nephron blockade, combining loop and thiazide diuretics, overcomes diuretic resistance, forces diuresis, and ameliorates acute congestive-related clinical outcomes. But association of two different classes of diuretics considerably increases the risks of electrolyte imbalance and renal function impairment, underscoring that decongestion alone does not suffice to reverse pathologic remodeling [54].

β-adrenergic blockers interrupt deleterious sympathetic overdrive, a hallmark of HF and adrenergic activation in obesity-related CV diseases [55]. These agents reduce myocardial oxygen demand by decreasing heart rate and contractility, improve ventricular filling times, and counteract arrhythmogenic triggers, all factors associated with CV diseases and worse clinical outcomes [56,57]. These agents also showed to positively modulate neurohormonal stress that drives myocardial remodeling in HF [58], and adjunctive effect explaining relevant amelioration of CV hard endpoints, including all-cause mortality, in both patients with HFpEF [59] and HFrEF [60]. Clinical trials lack outcomes stratified by BMI, significantly affecting proper assessment of β-adrenergic blocker safety and efficacy in patients with excess adiposity. From a practical view-point, it should be mentioned that β-adrenergic blockers are associated with a slight but not negligible weight gain and insulin sensitivity impairment, which are common side effects of this class of medications [61]. Moreover, β-blockade contributes less directly to regression of cardiomyocyte hypertrophy compared to renin-angiotensin system (RAAS) blockade.

The RAAS is upregulated in patients with obesity, leading to overproduction of aldosterone [62]. Angiotensin-converting enzyme (ACE) inhibitors and angiotensin receptor blockers are cornerstone therapies in CV diseases, since these attenuate maladaptive RAAS activation, reduce cardiac afterload and preload, and diminish angiotensin II-mediated profibrotic signaling [63]. Beyond lowering blood pressure, ACE inhibition has been repeatedly shown to facilitate regression of ventricle mass and improve endothelial function [64]. In patients with HF, ACE inhibitors reduce CV mortality by ~24% [65] and HF-related adverse outcomes by ~11% with high dose performing better than low dose [66].

Calcium-channel blockers (CCBs) exhibit antihypertensive activity via L-type voltage-gated calcium-channel antagonism, leading to arterial vasodilatation and reduced myocardial oxygen demand [67]. Long-acting dihydropyridine CCBs have demonstrated favorable effects on left ventricle hypertrophy reversal [68] and may reduce stroke risk in patients with arterial hypertension [69]. However, their direct impact on maladaptive myocardial remodeling in HF is generally less potent than that achieved through RAAS inhibition [70], especially in patients with preserved or mildly reduced ejection fraction (such as those with obesity-related HF) [71]. Non-dihydropyridine CCBs should instead be avoided in case of systolic dysfunction, since they exert negative inotropic effects prompting abrupt ejection fraction drops and acute decompensation [72,73].

Steroidal mineralocorticoid receptor antagonists (MRAs)—also defined as aldosterone antagonists—include spironolactone and eplerenone. These agents block aldosterone binding at the mineralocorticoid receptor and suppress the expression of epithelial sodium channels at the distal renal tubule, myocardium, and microvasculature, limiting sodium retention and attenuating aldosterone-induced hypertension, myocardial fibrosis, and vascular inflammation [74,75]. Landmark trials established that spironolactone reduces morbidity and hospitalization risk (−35%) and mortality (−30%) in patients with severe HF, substantiating the class as a critical component of guideline-directed medical therapy [76]. MRAs also provide anti-fibrotic benefits that complement those of ACE inhibitors and, partially, β-blockers by directly antagonizing aldosterone-mediated extracellular matrix deposition and pathological remodeling [77,78]. This effect is observed in patients with obesity, without HF, in which MRAs utilization reduces biomarkers of fibrosis and normalizes estimated cardiac filling pressures [79]. However, benefits are lower in patients with HFpEF, including obesity-related HF. Although the evidence indicates significant improvement in diastolic function [80] and positive results in terms of prevention of new or recurring hospital admissions due to HF decompensation [81], MRAs have negligible effects on CV mortality and other hard endpoints [82].

Recent pharmacological innovation has produced non-steroidal MRAs, most notably finerenone, which offers high receptor selectivity and a more favorable safety profile relative to classic steroidal MRAs [83]. Preclinical studies elucidate that finerenone mitigates oxidative stress, inflammation, and tissue fibrosis in myocardium and vasculature by blocking aldosterone-driven signaling cascades within cardiomyocytes, vascular smooth muscle cells and inflammatory cells [84]. Crucial trials—such as the FIDELIO-DKD [85], FIGARO-DKD [86], and FINEARTS-HF [87] in high-risk populations with T2D, chronic kidney disease, and HF–demonstrate that finerenone, on top of standard care (maximal tolerated ACE inhibitor or ARBs dose) significantly reduces HF hospitalization risk and all-cause mortality, with consistent benefits observed across a wide range of ejection fractions and metabolic profiles. Moreover, emerging evidence suggests that finerenone protective effects on CV events are independent of body weight, indicating its potential value in patients with excess adiposity, counteracting aldosterone excess and related maladaptive remodeling [88]. These findings position finerenone as an important adjunctive agent in current cardio-renal therapeutic paradigms, particularly for patients with obesity-associated HF and a high cardiometabolic risk burden.

Last, angiotensin receptor and neprilysin inhibitors (ARNI), such as sacubitril/valsartan, represent another strongly recommended pillar of the pharmacological treatment of HF, particularly HFrEF [89,90]. Patients with obesity overexpress neprilysin in the kidney and adipocytes, leading to myocardial injury and ventricular dysfunction [91]. As preclinical evidence suggests, neprilysin inhibition leads to additional benefits in obesity-related metabolic heart diseases, with improvements in left ventricular diastolic function, interstitial fibrosis, and oxidative stress [92]. As aforementioned, the PARADIGM trial demonstrated superior effects of sacubitril/valsartan than enalapril in reducing the risk of HF hospitalizations and mortality—with additional benefits on symptoms and quality of life in patients with HFrEF—and a further post hoc analysis revealed that sacubitril/valsartan reduced the risk of HF hospitalization or CV death across all BMI categories [93].

Together, these pharmacological classes—while differing in direct impact on fluid balance or neurohormonal axes—converge on a common goal: alleviating hemodynamic stress, preventing deleterious remodeling, and improving clinical outcomes in populations burdened by obesity-related CV diseases. Integrated use of decongestive and neurohormonal modulation remains the empiric standard in contemporary management, with ongoing research refining our understanding of how these interventions interact with obesogenic metabolic states. However, strong evidence of their beneficial effects in patients with obesity and HF remains largely underexplored, requiring further investigations.

## 6. Anti-Obesity Interventions and Heart Failure Outcomes

One of the most immediate and clinically relevant benefits of anti-obesity interventions in HFpEF is intentional weight reduction. Modest weight loss (e.g., 5% of baseline body weight) directly lowers cardiac workload by decreasing total blood volume, arterial pressure, left ventricular filling pressures, and ultimately reducing clinical signs and symptoms [94]. Closely related to weight reduction is the attenuation of pulmonary and systemic fluid retention, a hallmark of symptomatic HF [95].

Anti-obesity therapies—particularly those with natriuretic or osmotic diuretic properties—can reduce interstitial and intravascular congestion, thereby improving symptoms and exercise tolerance. This effect is clinically meaningful even in the absence of large changes in body weight and may contribute to reductions in HF decompensation [96].

Beyond hemodynamics, growing evidence supports anti-remodeling effects on the myocardium of weight loss. In fact, obesity is associated with concentric left ventricular hypertrophy, myocardial fibrosis, and impaired myocardial energetics and interventions aimed to improve metabolic efficiency and reduce lipotoxicity may partially reverse these structural and functional abnormalities [97]. Moreover pharmacological intervention—as reported with dapagliflozin—lead to significant shrinkage of EAT, independently of changes in BMI, and left ventricular mass compared to placebo in 12 months [98]. In this context, improvements in left ventricular mass, diastolic parameters, and biomarkers of myocardial stress observed in recent trials suggest that targeting obesity and metabolic dysfunction can modify the myocardial substrate itself, rather than alleviating symptoms.

Some anti-obesity and cardiometabolic therapies exert direct cardioprotective effects that are largely independent of weight loss (Table 6). For instance, sodium–glucose cotransporter 2 inhibitors (SGLT2i)—although inducing only modest body weight loss—consistently reduce HF-related outcomes, with most of the evidence derived from T2D [99,100,101]. SGTL2i mechanisms of benefit include natriuresis with preferential interstitial fluid removal, which is one of the first mechanisms of cardiac preload and afterload reduction, improvement in myocardial energetics through enhanced free-fatty acid and ketone bodies rather than glucose utilization, attenuation of inflammation and oxidative stress, and favorable effects on renal–cardiac interactions [102]. In patients with HFpEF, dapagliflozin reduces resting and exercise pulmonary capillary wedge pressure, along with favorable effects on plasma volume and body weight [103]. Based on clinical findings, in one trial, dapagliflozin was demonstrated to significantly reduce the main study outcome (worsening HF or CV death) across all weight classes: −26% in under- and normal-weight, −19% in overweight, −32% in class I obesity, −29% in class II and III obesity [104]. In HFrEF, the pivotal DAPA-HF trial established the prognostic efficacy of dapagliflozin in patients with ejection fraction ≤ 40%, demonstrating a significant reduction in the composite endpoint of worsening HF (hospitalization or urgent visit requiring intravenous therapy) by 30%, or CV death by 18%, irrespective of T2D [105]. Similarly, the EMPEROR-Reduced trial confirmed that empagliflozin significantly lowered the risk of CV death or HF hospitalization (−25%) in patients with symptomatic HFrEF, with a particularly consistent reduction in recurrent hospitalizations (−30%) [106]. Although primarily designed as CV outcome trials in diabetes, the CANVAS Program provided early signals of reduced HF hospitalization with canagliflozin [107], findings later reinforced in dedicated HF populations by the pragmatic CHIEF-HF trial, which demonstrated significant improvement in HF-related health status, assessed with change in the Kansas City Cardiomyopathy Questionnaire Total Symptom Score (KCCQ TSS) at 12 weeks (+4.3 points) across wide EF spectrum [108]. In EMPEROR-Preserved, empagliflozin significantly reduced the composite of CV death or HF hospitalization in patients with HFpEF (EF > 40%), irrespective of T2D, largely driven by low events of HF hospitalizations than placebo (−27%) [109]. Similar results were found in the EMPA-REG OUTCOME trial, in which empagliflozin utilization was associated with significant improvement of HF events regardless of baseline renal injury [110].

The DELIVER study demonstrated that dapagliflozin reduced worsening HF (−21%) and CV death (−12%) in patients with HFpEF, confirming a class effect of SGLT2i in HFpEF [111]. These findings were further strengthened by pooled analyses of EMPEROR trials, which showed a consistent treatment effect of empagliflozin across the continuum of EF (from <25% to 65%) reducing the cumulative risk of HF hospitalization by 30% [112]. Sotagliflozin, a dual SGLT1/2 inhibitor, provided complementary evidence in high-risk populations. The SOLOIST-WHF trial enrolled patients with T2D and recent worsening HF and demonstrated a significant reduction in CV death and total HF events [113] when sotagliflozin was initiated early (before or a few days after discharged), including in patients with HFpEF (−33%) [114]. The broader SCORED trial, conducted in patients with T2D and chronic kidney disease, also showed a relevant reduction in HF events—estimated as admission to hospital care or requirement of urgent visits for decompensated HF—by 33% [115]. In VERTIS cardiovascular trial, ertugliflozin reduced the risk of HF events by 30%, but most of the protection was observed in overweight individuals rather than patients with obesity [116]. Meta-analysis integrating data from DAPA-HF, DELIVER, EMPEROR-Reduced, EMPEROR-Preserved, SOLOIST-WHF confirmed a significant reduction in HF hospitalization events (−28%), as well as in total HF events (−23%), with benefits observed irrespective of baseline diabetes and across the EF spectrum [117]. Despite improvements in HF outcomes with SGLT2i are considered as a class effect, one head-to-head trial found empagliflozin more than dapagliflozin to produce better results in terms of all-cause mortality or hospital admission due to HF (−10%) [118].

A substantial body of randomized controlled trials and subsequent meta-analyses indicates that glucagon-like peptide 1 receptor agonists (GLP-1RA) not only produces robust and sustained weight loss in individuals with obesity, but also impacts clinical outcomes relevant to HF, particularly in patients with obesity-related HFpEF. One meta-analysis encompassing patients with obesity and HFpEF demonstrated that GLP-1RA significantly reduced the incidence of HF-related events by 60%, improved patient-reported quality of life as measured by the KCCQ, enhanced functional capacity (six-minute walk test), and produced substantial weight loss compared to placebo [119]. Treatment with GLP-1RA (vs. placebo) decreased the risk of new-onset HF (−23%) and the composite of HF events or cardiovascular death (−18%) in a total of 52,752 participants without HF from six RCTs [120]. While this pooled evidence did not show a statistically significant reduction in CV mortality or all-cause mortality, the magnitude of reduction in HF-driven events and patient-centered outcomes underscores a therapeutic indication in this high-risk population [121]. Importantly, this risk reduction appeared to be independent of changes in glucose control and body weight and correlated more closely with the preventive effects on atherosclerotic CV diseases [122]. In the SELECT trial—a trial dedicated to people living with preexisting CV disease and overweight or obesity but without T2D—once-weekly 2.4 mg semaglutide (34-month exposure) lead to a slight reduction in the risk of composite HF endpoint (−18%), mostly driven by semaglutide protection against deaths due to CV causes [123]. However, the result of a tailored study—the STEP-HFpEF DM trial—patients with obesity-related HFpEF and T2D showed significant improvement in HF-related symptoms and physical limitations after 1 year of semaglutide treatment (2.4 mg once-weekly) versus placebo [124]. Similar results were found in a population with obesity-related HFpEF, regardless of T2D, in which semaglutide 2.4 mg/weekly improves symptoms, attenuates physical limitations, and exercise tolerance and induces greater weight loss than placebo [125,126,127]. Moreover, semaglutide utilization reduces the use of all types of diuretics as reported by a pooled analysis of the STEP-HFpEF and STEP-HFpEF-DM trials [128]. Over 52 weeks, semaglutide significantly attenuates left atrial remodeling and right ventricular enlargement, while improving indices of diastolic function (E-wave velocity, E/A ratio, and E/e′), without affecting left ventricular dimensions or systolic function. These effects are independent of baseline T2D or atrial fibrillation, and greater weight loss is associated with greater reduction in left atrial volume [129]. Cumulatively, across four trials—SELECT, FLOW, STEP-HFpEF, and STEP-HFpEF DM (semaglutide 2.4 mg once-weekly in all, except for FLOW trial in which patients were treated with semaglutide 1 mg/week)—participants with HFpEF showed significant reduction in CV deaths or HF-related events by 31% and worsening HF events by 41% [130]. Lastly, prespecified analyses of the SOUL trial found that oral semaglutide was associated with a modest but significant 14% lower risk of the composite cardiovascular outcome, including worsening HF events, versus placebo, and this benefit was consistent regardless of concomitant SGLT2i use [131].

Mechanistically, GLP-1RA act by enhancing glucose-dependent insulin secretion, slowing gastric emptying, and promoting satiety through central nervous system pathways, resulting in profound reductions in body weight and adiposity. In patients with obesity and HFpEF, these metabolic effects are associated with improvements in diastolic function, reductions in systemic inflammation [132], and favorable changes in cardiac structure and function [133].

While direct evidence from HF trials remains limited compared with SGLT2i, especially in T2D, the cumulative evidence from RCTs and meta-analyses supports a role for GLP-1RA as a cardiometabolic intervention to reduce HF events, improves functional capacity and quality of life, and contributes in attenuating disease progression in patients with excess weight and obesity-related HFpEF.

Combining GLP-1RA to SGLT2i is expected to produce better results on several CV outcomes, including HF events. In a cohort study, patients receiving a GLP-1RA (particularly dulaglutide and semaglutide) and SGLT2i had significantly lower risk of mortality or hospital admission (composite −22%; mortality only −28%; hospital admission only −22%) and HF exacerbations (−23%) compared to SGLT2i alone [134]. These data suggest that additive/synergistic effects of GLP-1RA and SGLT2i co-administration could be useful instrument of treatment implementations in clinical practice.

Among novel anti-obesity agents, tirzepatide—a dual GLP-1 and glucose-dependent insulinotropic polypeptide (GIP) agonist—provided sufficient evidence on weight loss and cardiovascular protection. A post hoc analysis from the SURMOUNT-1 trial predicted that tirzepatide treatment is associated with a reduction in the risk of worsening HF by 46% relative to placebo in patients with obesity-related HFpEF [135]. The Tirzepatide effect was consistent in individuals with impaired renal function who exhibit baseline worse clinical presentation and progression of HFpEF compared to those with renal function within normal range [136]. Similar results have been reported in patients with obesity and T2D, where tirzepatide, compared with placebo, reduced the risk of the composite outcome of cardiovascular death or worsening HF by 38%, mostly attributable to fewer worsening HF events [137]. Tirzepatide, compared to placebo, produces a consistent beneficial effect across all composites of death and worsening heart failure events—analyzed as time to first event (hazard ratios, HRs = 0.41–0.62) [138]—and ameliorated the KCQCSS by 6.9 points, 6 min walk distance by 18.3 m, and EQ-5D-5L by 0.06 [139]. Tirzepatide also reduces circulatory volume–pressure overload and systemic inflammation, and mitigates cardiovascular-kidney end-organ injury in patients with obesity-related HFpEF [140]. Mechanistic data, moreover, indicates that treatment with tirzepatide decreases LV mass by 11 g, paracardial adipose tissue by 45 mL with both changes highly related to BMI and waist circumference reductions [141].

## 7. Conclusions and Future Perspective

Obesity-driven HF should be recognized as a distinct cardiometabolic entity rather than a mere coexistence of excess weight and cardiac dysfunction and its most common clinical presentation is HFpEF. Visceral and epicardial adiposity significantly contribute to myocardial remodeling, microvascular dysfunction, inflammation, and diastolic impairment, ultimately leading to elevated filling pressures and exercise intolerance despite preserved EF [142]. Efforts should be provided to prompt recognize and manage patients with obesity-related HF.

From a therapeutic standpoint, treating congestion alone addresses the hemodynamic expression of the disease without modifying its upstream drivers. Targeting the adipose–myocardial axis offers, instead, a therapeutic strategy to early intercept the pathogenic cascade linking excess adiposity to HFpEF. Sustained loss of visceral and epicardial fat depots has the potential to improve ventricular compliance, reduce inflammatory signaling, enhance metabolic efficiency of cardiomyocytes, and restore cardiorespiratory fitness.

For decades, HF therapy has focused on neurohormonal blockade and decongestion, approaches that remain indispensable. However, novel agents nowadays are widely available and, among them, SGLT2i have established consistent reductions in HF hospitalization across the EF spectrum, including HFpEF, as demonstrated in EMPEROR-Preserved and DELIVER. Data from clinical trials and real-word evidence reinforces their role as foundational therapy in HF. 

GLP-1RA and tirzepatide have shown substantial improvements in symptoms, functional capacity, and HF-related events in obesity-driven HFpEF populations—notably in STEP-HFpEF and cardiovascular outcomes in SELECT–with emerging reverse-remodeling signals from the SURMOUNT-1 trial. Compared to SGTL2i, GLP-1RA ensure more considerable weight loss, which is a therapeutic key in patients with excess weight; however, dedicated, large-scale, HF trials powered for hard CV endpoints and cardiac hemodynamics are needed to confirm whether incretin-based therapies can modify long-term prognosis in established HFpEF. Combination regimens integrating SGLT2i with incretin-based treatment warrant prospective evaluation, given their complementary hemodynamic, metabolic, and anti-inflammatory effects [143].

In conclusion, integrating structured weight management, currently approved pharmacologic anti-obesity strategies, and cardiometabolic therapies into a unified treatment pathway represents the best management of obesity-related HF [144].

## Figures and Tables

**Table 1:** Interplay Between Obesity and Cardiovascular Disease—Mechanisms and Pathophysiology [3].

Domain	Mechanisms/Pathways	Key Effects on Cardiovascular System
Adipose tissue as endocrine organ	↑ Adipokine (leptin, resistin) and pro-inflammatory cytokine (TNF-α, IL-6, MCP-1) secretion ↓ Anti-inflammatory adiponectin secretion EAT create a local pro-inflammatory milieu adjacent to vasculature/myocardium	Chronic systemic and vascular inflammation Local paracrine effects on coronary vessels and myocardium promoting atherosclerosis and microvascular dysfunction
Endothelial dysfunction	ROS overproduction and oxidative stress impair eNOS and reduce NO bioavailability Dysregulated adipokines contribute directly to endothelial injury PVAT dysfunction impairs anti-contractile effects on vessels (vasoconstriction)	Impaired endothelium-dependent vasodilation Early step in atherogenesis and progression to plaque formation Predicts arterial hypertension and future CV events
Atherogenesis	Chronic inflammation recruits monocytes → foam cells and plaque formation Dyslipidemia (↑ triglycerides, ↑ small dense LDL, ↓ HDL) accelerates lipid deposition in intima Oxidized LDL and inflammatory signaling drive plaque instability	Progressive atherosclerotic lesion development Plaque vulnerability and rupture risk Increased risk of MI and stroke
Hemodynamic stress and cardiac remodeling	Increased blood volume and cardiac output in obesity → hemodynamic overload RAAS and sympathetic activation (partially leptin mediated) raise blood pressure and promote sodium retention EAT expansion can mechanically restrict ventricular relaxation	LVH Diastolic dysfunction and HFpEF Progression to heart failure
Metabolic comorbidities	Insulin resistance and T2D, which further promote dyslipidemia and endothelial injury Sleep apnea induces intermittent hypoxia and sympathetic activation, worsening endothelial dysfunction	Synergistic amplification of CVD risk Elevated glucose levels and IR independently injure vasculature
Pro-thrombotic state	Obesity increases pro-coagulant factors and reduces fibrinolysis efficiency Adipose tissue secretes factors increasing platelet activation and clot formation	Higher risk of thrombosis, embolism, and acute coronary events
Traditional risk factor amplification	Obesity amplifies or causes: arterial hypertension, dyslipidemia, hyperglycemia, and physical inactivity	Additive and multiplicative effects on CVD risk Each factor independently contributes to incidence and severity of CVD outcomes

The table summarizes main pathophysiological pathways linking obesity to cardiovascular disease. Excess adiposity acts as an active endocrine and inflammatory organ that promotes endothelial dysfunction, atherogenesis, hemodynamic overload, metabolic derangements, and a pro-thrombotic state. These interconnected mechanisms synergistically amplify traditional cardiovascular risk factors and drive progression toward myocardial remodeling, heart failure, atherosclerotic events, and overt cardiovascular disease. Abbreviation list: CVD, cardiovascular disease; EAT, epicardial adipose tissue; eNOS, endothelial nitric oxide synthase; HDL, high-density lipoprotein; HFpEF, heart failure with preserved ejection fraction; IL-6, interleukin-6; IR, insulin resistance; LDL, low-density lipoprotein; LVH, left ventricular hypertrophy; MCP-1, monocyte chemoattractant protein-1; MI, myocardial infarction; NO, nitric oxide; PVAT, perivascular adipose tissue; RAAS, renin–angiotensin–aldosterone system; ROS, reactive oxygen species; T2D, type 2 diabetes; TNF-α, tumor necrosis factor-alpha. ↑, increase; ↓, decrease.

**Table 2:** Pathophysiological Links Between Obesity, Adipose Tissue, and Cardiovascular Dysfunction [5,6,7,8,9,10,11,12,13].

Pathophysiological Domain	Key Mechanisms	Cardiovascular Consequences
Adipose tissue as endocrine and immune organ	Expansion of visceral and ectopic adipose tissue induces chronic low-grade inflammation and dysregulated adipokine secretion	Systemic inflammatory burden contributing to endothelial dysfunction and myocardial injury
Inflammation and oxidative stress	Increased production of ROS, activation of inflammatory pathways (NF-κB, inflammasome), reduced nitric oxide bioavailability	Vascular homeostasis disruption, endothelial dysfunction, microvascular rarefaction
Perivascular adipose tissue dysfunction	Loss of anti-contractile properties, enhanced pro-inflammatory signaling	Increased vascular tone, arterial stiffness, impaired vasodilation
Metabolic derangements	Dyslipidemia (small dense LDL, hypertriglyceridemia), insulin resistance	Acceleration of atherosclerosis: lipid accumulation, plaque progression and instability
Hemodynamic alterations	Hyperdynamic circulation, hypervolemia (30% per 10 kg weight gain), increased preload and afterload	Chronic elevation of filling pressures, eccentric LV hypertrophy, diastolic dysfunction
Neurohormonal activation	Activation of sympathetic nervous system and renin–angiotensin–aldosterone system	Hypertension, myocardial remodeling, progression toward heart failure
Epicardial adipose tissue: inflammatory effects	Secretion of TNF-α, IL-6, IL-1β, MCP-1 by epicardial adipocytes, M1 macrophages, pro-inflammatory T-cells; reduced adiponectin expression	Local myocardial inflammation, extracellular matrix deposition, interstitial fibrosis
Epicardial adipose tissue: endothelial and microvascular effects	Paracrine release of inflammatory mediators and ROS impair coronary endothelial NO signaling	Coronary endothelial dysfunction, myocardial ischemia without obstructive CAD
Epicardial adipose tissue: mechanical effects	External compressive forces on the myocardium, reducing ventricular compliance	Impaired diastolic relaxation, elevated LV filling pressures, HFpEF phenotype
Atrial involvement	Epicardial fat infiltration and fibrotic remodeling causing structural and electrical heterogeneity	Increased susceptibility to atrial fibrillation and other supraventricular arrhythmias
Ectopic fat deposition and lipotoxicity	Ceramide and sphingolipid accumulation impair mitochondrial β-oxidation	Myocyte injury, energetic failure, adverse myocardial remodeling
Impaired cardiac regeneration	Disruption of cardiac progenitor cell niche and inhibited differentiation into cardiomyocytes	Reduced regenerative capacity, persistence of maladaptive remodeling

The table summarizes the integrated mechanisms linking obesity to myocardial remodeling and cardiovascular disease. Excess visceral and epicardial fat drives chronic inflammation, oxidative stress, endothelial and microvascular dysfunction, neurohormonal activation, and mechanical constraint of the myocardium. These processes converge toward adverse ventricular remodeling, diastolic dysfunction, atrial arrhythmogenesis, and progression to heart failure and overt cardiovascular disease. Abbreviation list: CAD, coronary artery disease; HFpEF, heart failure with preserved ejection fraction; IL-1β, interleukin-1 beta; IL-6, interleukin-6; LDL: low-density lipoprotein; LV, left ventricle/left ventricular; M1: classically activated macrophage phenotype; MCP-1, monocyte chemoattractant protein-1; NF-κB, nuclear factor kappa-light-chain-enhancer of activated B cells; NO, nitric oxide; ROS, reactive oxygen species; TNF-α, tumor necrosis factor-alpha.

**Table 3:** Clinical and Echocardiographic Features of Obesity-Associated Heart Failure [30,31,32,33].

Domain	Key Features	Clinical Implications
Epidemiological profile	Severe or long-standing obesity (often BMI ≥ 35 kg/m^2^); female predominance in HFpEF phenotype; frequent coexistence of arterial hypertension, T2D, and obstructive sleep apnea	High cardiometabolic burden; under-recognized HF due to symptom overlapping with obesity
Hemodynamic changes	Expanded plasma volume; increased cardiac output (high-output state in early phases); increased preload and afterload; arterial stiffness	Progressive ventricular remodeling; elevated filling pressures, especially during exertion
Neurohormonal and metabolic activation	RAAS activation; sympathetic overactivity; insulin resistance; chronic low-grade inflammation; adipokine dysregulation	Promotes hypertrophy, fibrosis, and microvascular dysfunction
Left ventricular structure	Concentric LV hypertrophy; increased LV mass index; relative wall thickness increased; normal or mildly reduced LV cavity size	Typical substrate of HFpEF phenotype
Left ventricular function	Preserved LVEF in most cases; impaired diastolic relaxation (Grade I-II dysfunction); elevated E/e′ ratio; reduced diastolic reserve	Elevated LV filling pressures, particularly with exercise
Left atrium	Enlarged left atrial volume index; impaired LA reservoir function	Reflects chronic elevation of filling pressures
Right heart and pulmonary circulation	Mild right ventricular dysfunction; increased pulmonary artery systolic pressure; functional tricuspid regurgitation in advanced stages	Contributes to exercise intolerance and congestion
Epicardial adipose tissue	Increased epicardial fat thickness; mechanical constraint and paracrine inflammatory effects	May impair ventricular compliance and promote fibrosis
Functional capacity	Reduced VO_2_ peak; chronotropic incompetence; impaired ventricular–vascular coupling; abnormal hemodynamic response to exercise	Disproportionate exertional dyspnea relative to resting findings
Biomarkers	Natriuretic peptides often lower than expected for degree of congestion; elevated CRP and inflammatory markers	Diagnostic challenge; risk of underestimating HF severity
Clinical phenotype	Predominantly HFpEF; high symptom burden; exercise intolerance; edema variable	Obesity-driven HF with preserved EF and elevated filling pressures

The table resumes the clinical and hemodynamic profile of obesity-driven heart failure, predominantly HFpEF. Patients typically present with severe or long-standing obesity, often complicated with arterial hypertension, type 2 diabetes, and sleep apnea. Key features include expanded plasma volume, concentric LV hypertrophy with preserved EF, diastolic dysfunction, left atrial enlargement, and subtle right heart involvement. Epicardial adipose tissue contributes mechanically and, mostly, via paracrine inflammation. Functional capacity is disproportionately reduced relative to resting findings, and natriuretic peptides may underestimate congestion, creating diagnostic challenges. Overall, this phenotype represents obesity-related HFpEF with elevated filling pressures and high cardiometabolic burden. Abbreviation list: BMI, body mass index; CRP, C-reactive protein; EF, ejection fraction; HF, heart failure; HFpEF, heart failure with preserved ejection fraction; LA: left atrium; LV, left ventricle/left ventricular; LVEF, left ventricular ejection fraction; RAAS, renin–angiotensin–aldosterone system; T2D, type 2 diabetes; VO_2_, oxygen consumption peak.

**Table 4:** Simplified Comparison Between HFA-PEFF and H2FPEF Scores [35,36,39].

Feature	HFA-PEFF Score	H2FPEF Score
Purpose	Structured diagnostic algorithm for HFpEF based on guideline criteria	Simple clinical score to estimate probability of HFpEF
General approach	Stepwise, multiparametric evaluation integrating imaging, biomarkers, and function	Point-based bedside tool focused on clinical characteristics
Main components	3 domains: Functional (diastolic indices), Morphological (LA size, LV mass), Biomarkers (natriuretic peptides)	6 variables: Obesity, ≥2 antihypertensive drugs, Atrial fibrillation, Pulmonary hypertension, Age > 60 years, Elevated filling pressures (E/e′)
Role of imaging	Central and detailed; requires comprehensive echocardiographic assessment	Limited; mainly relies on E/e’ and estimated pulmonary pressure
Role of natriuretic peptides	Formally included in the score	Not included
Scoring system	Major and minor criteria within each domain; intermediate results require further testing	Additive score (0–9 points); higher score = higher probability
Diagnostic pathway	Provides guidance for additional testing (stress echo or invasive hemodynamics) if indeterminate	Does not include a structured next-step algorithm
Overall diagnostic performance (general population)	Good diagnostic discrimination when full algorithm is applied; higher specificity in structured settings	Good discrimination and practical sensitivity in outpatient and referral cohorts
Limitations in obesity	Natriuretic peptide levels may be falsely low; echocardiographic image quality may be suboptimal due to adipose tissue attenuation	Obesity contributes directly to the score (2 points), potentially inflating probability independently of true diastolic dysfunction; absence of biomarker adjustment
Main strengths	Pathophysiology-based, comprehensive, guideline-aligned	Practical, rapid, easy to apply
Main limitations	Intricate; dependent on imaging expertise and biomarker interpretation	May overemphasize age and comorbidities; less structural characterization

The table briefly summarizes comparison between the HFA-PEFF and H2FPEF scores for the diagnosis of HFpEF. The table highlights their conceptual framework, core components (clinical variables, imaging parameters, and biomarkers), diagnostic workflow, overall performance in general populations, and phenotype-specific limitations, particularly in patients with obesity. Abbreviation list: HFpEF, Heart Failure with Preserved Ejection Fraction; HFA-PEFF, Heart Failure Association Pre-test Assessment Echocardiography and natriuretic Peptide, Functional Testing, Final Etiological Workup; H2FPEF, Heavy, Hypertensive, Atrial Fibrillation, Pulmonary Hypertension, Elder, Filling Pressure; LA, Left Atrium; LV, Left Ventricle; E/e′, Ratio of early transmitral Doppler velocity (E) to early diastolic mitral annular velocity (e′).

**Table 5:** Conventional Pharmacological Treatment of Heart Failure with Emphasis on Obesity-Related HFpEF: Summary of Mechanisms and Clinical Evidence [43,44,45,46,47,48,49,50,51,52,53,54,55,56,57,58,59,60,61,62,63,64,65,66,67,68,69,70,71,72,73,74,75,76,77,78,79,80,81,82,83,84,85,86,87,88,89,90,91,92,93].

Drug Class	Mechanism of Action	Effects on Hemodynamics and Remodeling	Evidence on Hard Endpoints	Evidence in Obesity/ Obesity-Related HFpEF	Key Limitations/Safety Concerns
Loop Diuretics	Inhibit Na^+^-K^+^-2Cl^−^ cotransporter in thick ascending limb → rapid natriuresis and diuresis	Rapid symptom relief; reduced preload and venous congestion; no direct anti-remodeling effect; compensative RAAS activation	Neutral or controversial long-term effects vs. neurohormonal therapies; high chronic doses associated with worse outcomes in HFpEF	Obese patients highly responsive to IV therapy; chronic high-dose exposure linked to excess CV mortality in HFpEF; increased risk of worsening renal function	RAAS activation; intrarenal plasma flow reduction; electrolyte imbalance; possible renal impairment
Thiazide/Thiazide-like Diuretics	Inhibit Na^+^-Cl^−^ cotransporter in distal convoluted tubule	Enhance residual sodium excretion; no structural remodeling effects	Mild or neutral long-term CV outcome impact in HFpEF	Used to overcome diuretic resistance (sequential nephron blockade); limited direct outcome data in obesity-related HF	Electrolyte imbalance; renal dysfunction when combined with loop diuretics
β-Adrenergic Blockers	Inhibit sympathetic overdrive; reduce HR and contractility → low oxygen consumption	Improve ventricular filling; reduce arrhythmogenic burden; partial anti-remodeling effects (lower than RAAS blockade)	Reduce all-cause mortality and CV events in HF (strong evidence in HFrEF; supportive in HFpEF)	No BMI-stratified RCT data; weight gain may occur; potentially beneficial in obesity-related sympathetic overactivation	Mild weight gain; limited regression of hypertrophy compared with RAAS inhibition
ACE Inhibitors or ARB	Inhibit conversion of angiotensin I to angiotensin II or block angiotensin receptor → reduce afterload, preload, and profibrotic signaling	Promote LV mass regression; improve endothelial function	Reduce CV mortality (~24%) and HF-related adverse outcomes (~11%); high dose superior to low dose	Particularly relevant in obesity with upregulated RAAS; limited obesity-specific RCT stratification	Hypotension; renal function impairment; hyperkalemia
Calcium-Channel Blockers (CCBs)	Block L-type calcium channels → arterial vasodilation	LV hypertrophy regression (dihydropyridines); limited impact on HF remodeling	Stroke reduction in hypertension; less effective than RAAS blockade in HF populations	Limited direct evidence in obesity-related HF; neprilysin/RAAS-targeting therapies superior	Non-dihydropyridines contraindicated in systolic dysfunction (negative inotropy)
Steroidal MRAs	Block aldosterone at mineralocorticoid receptor → reduce sodium retention, fibrosis, inflammation	Anti-fibrotic; complement ACEi and β-blockers; improve diastolic function	Reduce morbidity (−35%) and mortality (−30%) in severe HF; negligible mortality reduction in HFpEF	Reduce fibrosis biomarkers and filling pressures in obesity without HF; in HFpEF reduce hospitalizations but not mortality	Hyperkalemia; renal impairment; endocrine side effects (spironolactone)
Non-Steroidal MRA	Selective mineralocorticoid receptor antagonism; anti-inflammatory, anti-fibrotic	Attenuates oxidative stress and extracellular matrix deposition	In FIDELIO-DKD, FIGARO-DKD, and FINEARTS-HF: reduced HF hospitalization and all-cause mortality on top of standard care	Benefits consistent across metabolic profiles and independent of BMI; promising for obesity-associated HF	Hyperkalemia (lower incidence vs. steroidal MRA); cost considerations
ARNI	Neprilysin inhibition + AT1 receptor blockade → enhanced natriuretic peptides and RAS suppression	Reverse remodeling; improve diastolic function, reduce fibrosis and oxidative stress	In PARADIGM-HF: superior to enalapril in reducing HF hospitalization and CV mortality; benefits across BMI classes	Patients with obesity overexpress neprilysin; post hoc analyses show consistent benefit across BMI ranges	Hypotension; renal function monitoring required

The table summarizes major pharmacological therapies used in heart failure, with particular emphasis on mechanisms, remodeling effects, cardiovascular outcomes, and available evidence in obesity-related HFpEF. Abbreviations: ACE, angiotensin-converting enzyme; ARB, angiotensin receptor blocker; ARNI, angiotensin receptor neprilysin inhibitor; BMI, body mass index; CCBs, calcium-channel blockers; CV, cardiovascular; HF, heart failure; HFpEF, heart failure with preserved ejection fraction; HFrEF, heart failure with reduced ejection fraction; HR, heart rate; IV, intravenous; LV, left ventricle; MRAs, mineralocorticoid receptor antagonist; RAAS, renin–angiotensin–aldosterone system; RCT, randomized controlled trial.

**Table 6:** Effects of SGLT2i, GLP-1RA, Combination Therapy, and Tirzepatide in HFrEF and HFpEF [94,95,96,97,98,99,100,101,102,103,104,105,106,107,108,109,110,111,112,113,114,115,116,117,118,119,120,121,122,123,124,125,126,127,128,129,130,131,132,133,134,135,136,137,138,139,140,141].

Class	HFrEF	HFpEF	Strength of Evidence
SGLT2i	Significant ↓ CV death and ↓ HHF (~25–30%) in dedicated HFrEF trials (DAPA-HF; EMPEROR-Reduced). Benefits independent of T2D.	↓ HHF and worsening HF across the EF spectrum (EMPEROR-Preserved; DELIVER). Effect mainly driven by hospitalization reduction.	Robust, guideline-directed, disease-modifying therapy across the EF continuum.
GLP-1RA	No dedicated positive HFrEF outcome trials. CVOTs show ↓ incident HF and ↓ composite HF outcomes, but not powered for established HFrEF and related outcomes.	In obesity-related HFpEF: marked improvement in symptoms (KCCQ) and functional capacity (6MWD) (STEP-HFpEF), ↓ composite HF endpoints in high-risk populations (SELECT).	Strong evidence for symptom improvement and HF risk reduction in obesity-driven HFpEF; limited direct HFrEF data.
SGLT2i + GLP-1RA	Observational and cohort data suggest additive ↓ mortality and ↓ HHF vs. SGLT2i alone; no dedicated RCT in established HFrEF.	Mechanistically complementary (hemodynamic + metabolic). Cohort data suggest ↓ HF exacerbations vs. monotherapy; no dedicated HFpEF RCT.	Promising additive strategy; high biological plausibility; RCT evidence pending.
Tirzepatide (GLP-1/GIP dual RA)	No dedicated HFrEF outcome trial. Signals of ↓ worsening HF in obesity populations (post hoc analyses of SURMOUNT-1).	Significant weight loss; ↓ worsening HF risk; ↑ KCCQ and ↑ 6MWD in obesity-related HF phenotypes (post hoc and emerging data).	Emerging disease-modifying potential; confirmatory dedicated HF trials warranted.

The table summarizes comparative evidence of SGLT2i, GLP-1RA combination therapy, and tirzepatide across HFrEF and HFpEF phenotypes. SGLT2i demonstrate consistent hard outcome reduction across the EF spectrum. GLP-1RA and tirzepatide primarily improve cardiometabolic substrate, symptoms, and HF risk in obesity-related HFpEF, with limited direct HFrEF evidence. Combination therapy (GLP-1RA + SGLT2i) shows biologically complementary effects, but randomized HF-specific data are needed. Abbreviations: CV, cardiovascular; CVOT, cardiovascular outcomes trials; EF, ejection fraction; GLP-1RA, glucagon-like peptide-1 receptor agonists; GIP, glucose-dependent insulinotropic polypeptide; HF, heart failure; HFpEF, heart failure with preserved ejection fraction; HFrEF, heart failure with reduced ejection fraction; HHF, hospitalization for heart failure; KCCQ, Kansas City Cardiomyopathy Questionnaire; RCT, randomized controlled trial; SGLT2i, sodium–glucose cotransporter type 2 inhibitors; T2D: type 2 diabetes; 6MWD, 6 min walk distance. ↑, increase; ↓, decrease.

## Data Availability

No new data were generated or analyzed. Data sharing is not applicable to this paper.

## Abbreviations

ACE	Angiotensin-Converting Enzyme
ARB	Angiotensin Receptor Blocker
ARNI	Angiotensin Receptor Neprilysin Inhibitor
BMI	Body Mass Index
CANVAS	Canagliflozin Cardiovascular Assessment Study
CCBs	Calcium-Channel Blockers
CHIEF-HF	Canagliflozin: Impact on Health Status, Quality of Life and Functional Status in Heart Failure
CI	Confidence Interval
CPC	Cardiac Progenitor Cell
CRP	C-Reactive Protein
CV	Cardiovascular
CVD	Cardiovascular Disease
DAPA-HF	Dapagliflozin and Prevention of Adverse Outcomes in Heart Failure
DELIVER	Dapagliflozin Evaluation to Improve the Lives of Patients With Preserved Ejection Fraction Heart Failure
EAT	Epicardial Adipose Tissue
EF	Ejection Fraction
E/e′	Rapporto tra velocità del flusso transmitralico precoce (E) e velocità diastolica precoce dell’anello mitralico (e')
EMPA-REG OUTCOME	Empagliflozin Cardiovascular Outcome Event Trial in Type 2 Diabetes Mellitus Patients
EMPEROR-Preserved	Empagliflozin Outcome Trial in Patients With Chronic Heart Failure With Preserved Ejection Fraction
EMPEROR-Reduced	Empagliflozin Outcome Trial in Patients With Chronic Heart Failure With Reduced Ejection Fraction
EQ-5D-5L	EuroQol 5 Dimensions 5 Levels
FLOW	Effect of Semaglutide Versus Placebo on the Progression of Renal Impairment in Subjects With Type 2 Diabetes and Chronic Kidney Disease
FIGARO-DKD	Finerenone in Reducing Cardiovascular Mortality and Morbidity in Diabetic Kidney Disease
FIDELIO-DKD	Finerenone in Reducing Kidney Failure and Disease Progression in Diabetic Kidney Disease
FINEARTS-HF	Finerenone Trial to Investigate Efficacy and Safety in Heart Failure
HF	Heart Failure
HFA-PEFF	Heart Failure Association Pre-test Assessment, Echocardiography and Natriuretic Peptide Score
HFpEF	Heart Failure with Preserved Ejection Fraction
HFrEF	Heart Failure with Reduced Ejection Fraction
HR	Hazard Ratio
H2FPEF	Heavy, Hypertensive, Atrial Fibrillation, Pulmonary Hypertension, Elder, Filling Pressure Score
IL-1β	Interleukin 1 beta
IL-6	Interleukin 6
KCCQ	Kansas City Cardiomyopathy Questionnaire
KCCQ CSS	Kansas City Cardiomyopathy Questionnaire Clinical Summary Score
KCCQ TSS	Kansas City Cardiomyopathy Questionnaire Total Symptom Score
LDL	Low-Density Lipoprotein
MRAs	Mineralocorticoid Receptor Antagonists
PARADIGM	Prospective Comparison of ARNI with ACEI to Determine Impact on Global Mortality and Morbidity in Heart Failure
RAAS	Renin–Angiotensin System
RCT	Randomized Controlled Trial
SCORED	Sotagliflozin on Cardiovascular and Renal Events in Patients With Type 2 Diabetes and Moderate Renal Impairment
SELECT	Semaglutide Effects on Cardiovascular Outcomes in People With Overweight or Obesity
SGTL2i	Sodium–Glucose Cotransporter 2 Inhibitor
SGLT1/2	Sodium–Glucose Cotransporter 1 and 2
SOLOIST-WHF	Sotagliflozin in Patients With Diabetes and Recent Worsening Heart Failure
SOUL	Semaglutide cardiOvascular oUtcomes triaL
STEP-HFpEF	Semaglutide Treatment Effect in People With Obesity and HFpEF
STEP-HFpEF DM	Semaglutide Treatment Effect in People With Obesity and HFpEF and Type 2 Diabetes
SURMOUNT-1	Tirzepatide Once-Weekly for the Treatment of Obesity
TNF-α	Tumor Necrosis Factor-Alpha
T2D	Type 2 Diabetes
VERTIS	Evaluation of Ertugliflozin Efficacy and Safety Cardiovascular Outcomes Trial
VO_2_ max	Maximal Oxygen Consumption
WHR	Waist-to-Hip-Ratio
